# Long non-coding RNA XIST regulates ovarian cancer progression via modulating miR-335/BCL2L2 axis

**DOI:** 10.1186/s12957-021-02274-7

**Published:** 2021-06-05

**Authors:** Qingjuan Meng, Ningning Wang, Guanglan Duan

**Affiliations:** 1 Medical Examination Center, The Third Hospital of Jinan, Jinan, 250132 China; 2 Department of Obstetrics, Jinan Central Hospital, Cheeloo College of Medicine, Shandong University, Jinan, 250013 Shandong China; 3 Department of Urology Surgery, Jinan Central Hospital, Cheeloo College of Medicine, Shandong University, Jinan, 250013 Shandong China

**Keywords:** XIST, Ovarian cancer, BCL2L2, miR-335

## Abstract

**Background:**

X inactivation-specific transcript (XIST) is the long non-coding RNA (lncRNA) related to cancer, which is involved in the development and progression of various types of tumor. However, up to now, the exact role and molecular mechanism of XIST in the progression of ovarian cancer are not clear. We studied the function of XIST in ovarian cancer cells and clinical tumor specimens.

**Methods:**

RT-qPCR was performed to detect the expression levels of miR-335 and BCL2L2 in ovarian cancer cells and tissues. MTT and transwell assays were carried out to detect cell proliferation, migration, and invasion abilities. Western blot was performed to analyze the expression level of BCL2L2. The interaction between miR-335 and XIST/BCL2L2 was confirmed using a luciferase reporter assay.

**Results:**

The inhibition of XIST can inhibit the proliferation invasion and migration of human ovarian cancer cells. In addition, the miR-335/BCL2L2 axis was involved in the functions of XIST in ovarian cancer cells. These results suggested that XIST could regulate tumor proliferation and invasion and migration via modulating miR-335/BCL2L2.

**Conclusion:**

XIST might be a carcinogenic lncRNA in ovarian cancer by regulating miR-335, and it can serve as a therapeutic target in human ovarian cancer.

## Introduction

Ovarian cancer has the highest lethal rate among gynecological tumors, and it is the fifth leading cause of cancer mortality in women. Because of its occult onset, it is diagnosed late in 70% of cases [1]. The prognosis is poor, and the 5-year survival rate is relatively low, mainly due to the extensive invasion and metastasis of the tumor. To explore which factors play a significant part in the invasion and migration of ovarian cancer, it is crucial to study the specific mechanism of promoting tumor development.

MicroRNAs (miRNAs) are endogenous and evolutionarily conserved small non-coding RNAs that regulate gene expression through base pairing with target mRNA [2]. miRNA plays an important role in regulating many biological processes, including proliferation, differentiation, and apoptosis [3, 4]. Recent evidence shows that miRNA can play a part in inhibiting or promoting cancer development [4]. Unusual expression of miRNA has been observed in breast cancer, ovarian cancer, and pancreatic cancer, which indicates that they may be useful in clinical diagnosis and treatment of cancer [5–8].

miR-335 has been found to play an oncogene or tumor suppressor role in various kinds of cancers [9]. It can inhibit the metastasis of breast cancer cells [10]. In the meanwhile, miR-335 was discovered to inhibit the metastasis of gastric cancer in vivo [11]. Lynch et al. [12] also found that miR-335 inhibited the invasion and migration latent of neuroblastoma cells. Amusingly, miR-335 has been discovered to be upregulated in glioma [13]. It is a putative miRNA oncogene, which can give malignant astrocytoma cell lines carcinogenic characteristics such as tumor growth and invasion in vitro. It may be a potential need for the transformation of malignant glioma cell lines [14]. These outcomes state clearly that the role of miR-335 varies depending on the cancer type. Previous studies have linked miR-335 to ovarian cancer [15–17]. Nevertheless, its expression and action remain to be explored in ovarian tumors. Using an online prediction algorithm to hunt for miR-335 control targets that may involve in ovarian cancer proceeding, BCL2L2 has been identified, which has a supposed miR-335 combining site in 3′UTR.

BCL2 family protein is an important regulator of the programmed cell death pathway of a single member. Its expression can inhibit or promote apoptosis [18]. The expression of BCL2 family members in cancer cells may be a useful indicator of disease responsiveness. For example, BCL2L2 (protein 2 similar to BCL2, also known as BCL-w), myeloid leukemia 1, and BCL2 are observed to be overexpressed in various kinds of cancers, leading to carcinogenesis and apoptosis inhibition [19–21]. It could boost cell invasion in gastric cancer [22] and restrain cell death in colorectal cancer [23]. In addition, it has been reported that the response to apoptosis is a response to a variety of physiological cues and cytotoxic agents, controlled by proteins of the BCL2 family [18]. Therefore, targeting the gene family may provide a new way to inhibit cancer development and induce tumor cell apoptosis.

Recent studies have shown that long non-coding RNA (lncRNA) is associated with the normal development of diseases, including cancer [24]. LncRNA has become a new participant in cancer study and treatment. Various studies have suggested that lncRNAs play crucial roles in different tumors [25]. The interaction between lncRNA and microRNA plays a principal part in tumorigenesis [26, 27]. Zhu et al. found that the lncRNA H19/miR-675 axis can inhibit the transforming growth factor β-inducible protein (TGF-β1) [28] of prostate cancer through targeted transformation. LncRNA-H19 was found to regulate the proliferation and migration of gastric cancer cells [29]. The XIST transcripts are products of the XIST gene, which is highly expressed and drives tumorigenesis in non-small cell lung cancer [30]. However, the exact molecular mechanism of XIST in ovarian cancer remains unclear. In this study, we reported the interaction between XIST and miR-335, which regulates the growth of ovarian cancer cells by targeting BCL2L2 directly. Our findings provided a novel understanding of the role of XIST and miR-335 in ovarian cancer metastasis and its related mechanisms.

## Materials and methods

### Tissue samples

From 2004 to 2009, 30 cases of ovarian cancer tissue samples were obtained from patients undergoing surgery at the Third Hospital of Jinan. Normal tissue was obtained from ovarian wedge biopsy or adnexectomy for fibroids or adenomyosis. After ovariectomy, the germinal epithelium was removed and further analyzed. This study was approved by the ethics committee of the Third Hospital of Jinan, and all patients provided written informed consent.

### Cell culture and transfection

Human epithelial ovarian cancer cell lines SKOV3 and OVCAR3 were purchased from China Typical Culture Preservation Center (Wuhan, China), and 10% FBS (GIBCO BRL, USA) was supplemented in Dulbecco modified Eagle medium (DMEM) (GIBCO BRL, USA). For the overexpression of miRNA-335, SKOV3 and OVCAR3 cells were transfected with miR-335 mimic (GenePharma, China). For the overexpression of BCL2L2, SKOV3 cells were transfected with the BCL2L2 expression construct, which has become insensitive through the mutation of 3′UTR (GenePharma, USA) of miR-335. Lipofectamine was used in all transfections Gamma 2000 reagents (Invitrogen life technologies, USA) which are carried out according to the manufacturer’s instructions.

### RNA extraction and real-time PCR analysis

The total RNA was extracted by TRIzol Reagent (Invitrogen, USA). Following the manufacturer’s instructions, the Hairpin-it Gamma MiRNA qPCR quantitative Kit (GenePharma, China) was used for analyzing the miR-335. U6 is used as a reference gene for standardization. The 25 μl PCR mixture consisted of 2× SYBR Green qPCR main mixture (Toyobo, Japan). The primers for BCL2L2 are as follows: forward 5′-CTT GGT CTT GTT GTG AGT ATG C A-3′ and reverse 5′-TGG AGC CGA TGG TCT AGT C-3; miR-335, forward 5′-UGU UUU GAG CGG GGG UCA AGA GC-3′ and reverse 5′-CUC UCA UUU GCU AUU CA-3′; and β-actin, forward 5′-GCC AAC AGT GCT GTC TGG-3′ and reverse 5′-GCT CAG GAG AGC AAT GAT CTT G-3′. β-Actin is used as a standardized reference gene. The relative expression was calculated by the 2^−ΔΔCT^ method.

### Dual-luciferase assay

The 3′UTR of wild-type or mutant BCL2L2 was inserted into the pmirGLO (Promega, USA). Hek-293t cells were co-transfected with the plasmids of wild-type or mutant BCL2L2 and miR-335. Forty-eight hours after transfection, firefly luciferase activity/renilla luciferase activity was evaluated by a dual-luciferase reporter gene analysis system (Promega, China).

### MTT assay

Transwell chamber (Corning, USA) was used to evaluate the migration and invasion ability of ovarian cells. 5 × 10^4^ cells were placed in the uncoated upper chamber without serum, and the lower chamber was full of 10% fetal bovine serum (FBS) to induce ovarian cell migration or invasion of the membrane. In addition, cells were put in the upper chamber together with the coated membrane for invasion assay. These cells were cultured for 48 h or 72 h for migration and invasion assay. Cells were then stained with crystal violet (Beyotime, Shanghai, China).

### Western blot analysis

Total protein in cultured cells was dissolved in RIPA buffer (Sigma, Japan) and used the BCA protein assay kit (Beyotime, China). Then, the proteins were electrophoresed on SDS-PAGE and transferred to the PVDF membranes (Millipore, USA). The membranes were diluted with 5% skimmed milk powder in TBST for 1 h at room temperature and then overnight at 4 °C with primary antibody. The membrane was then incubated for 2 h at room temperature with the corresponding concentration of diluted secondary antibody. The integrated density quantifies the number of proteins using the ImageJ software (NIH).

### Statistical analysis

The data were expressed as one of three independent experiments with mean ± SD, and the SPSS 17.0 statistical software was used (SPSS, Inc., Chicago, IL, USA). Using Wilcoxon’s paired test, we compared miR-335 in ovarian cancer and matched normal tissue. One-way ANOVA was used to evaluate the differences between the groups. P < 0.05 is considered statistically significant.

## Results

### miR-335 negatively regulated the expression of BCL2L2 in SKOV3 and OVCAR3 cell lines

**Fig. 1:**
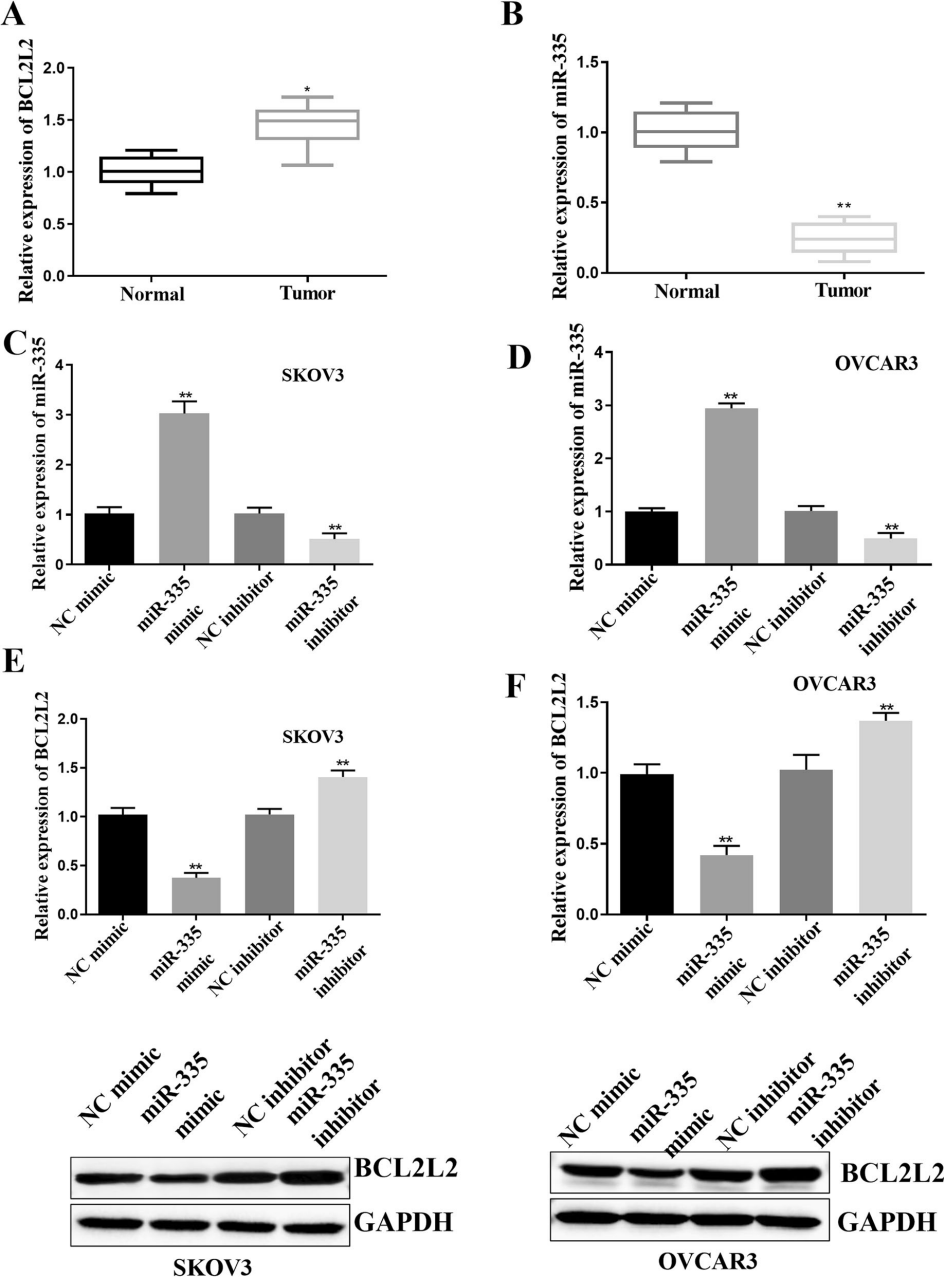
Negative correlation between miR-335 and BCL2L2 levels in ovarian cancer. **A** BCL2L2 levels in ovarian cancer tissues were significantly upregulated. **B** The levels of miR-335 in ovarian cancer tissues were significantly decreased. **C**, **D** miR-335 mimic was used to achieve overexpression of miR-335, while miR-335 inhibitor was used to achieve inhibition of miR-335 in SKOV3 and OVCAR3 cell lines. **E**, **F** miR-335 overexpression inhibited while miR-335 silence increased the expression of BCL2L2 in SKOV3 and OVCAR3 cell lines. *P < 0.05, **P < 0.01

Quantitative PCR analysis was performed to analyze the expression level of miR-335 in ovarian cancer quantitatively. In ovarian cancer and adjacent normal tissues, the expression of miR-335 in ovarian cancer tissues was significantly lower than that in adjacent normal tissues (*p*<0.05, Fig. 1A). On the contrary, BCL2L2 was upregulated in ovarian cancer compared to matched adjacent normal tissues (*p*<0.01, Fig. 1B). The SKOV3 and OVCAR3 cells were transfected with miR-335 mimic and the miR-335 inhibitor to explore the effects of miR-335 on ovarian cancer progression (*p*<0.01, Fig. 1C, D). Next, in response to the overexpression of miR-335 or inhibition of miR-335, qRT-PCR and Western blotting were used to determine the expression of BCL2L2. The results showed that the overexpression of miR-335 inhibited the expression of BCL2L2, while the inhibition of miR-335 promoted the expression of BCL2L2 in ovarian cell lines (*p*<0.01, Fig. 1E, F).

### BCL2L2 mRNA was the direct target of miR-335

**Fig. 2:**
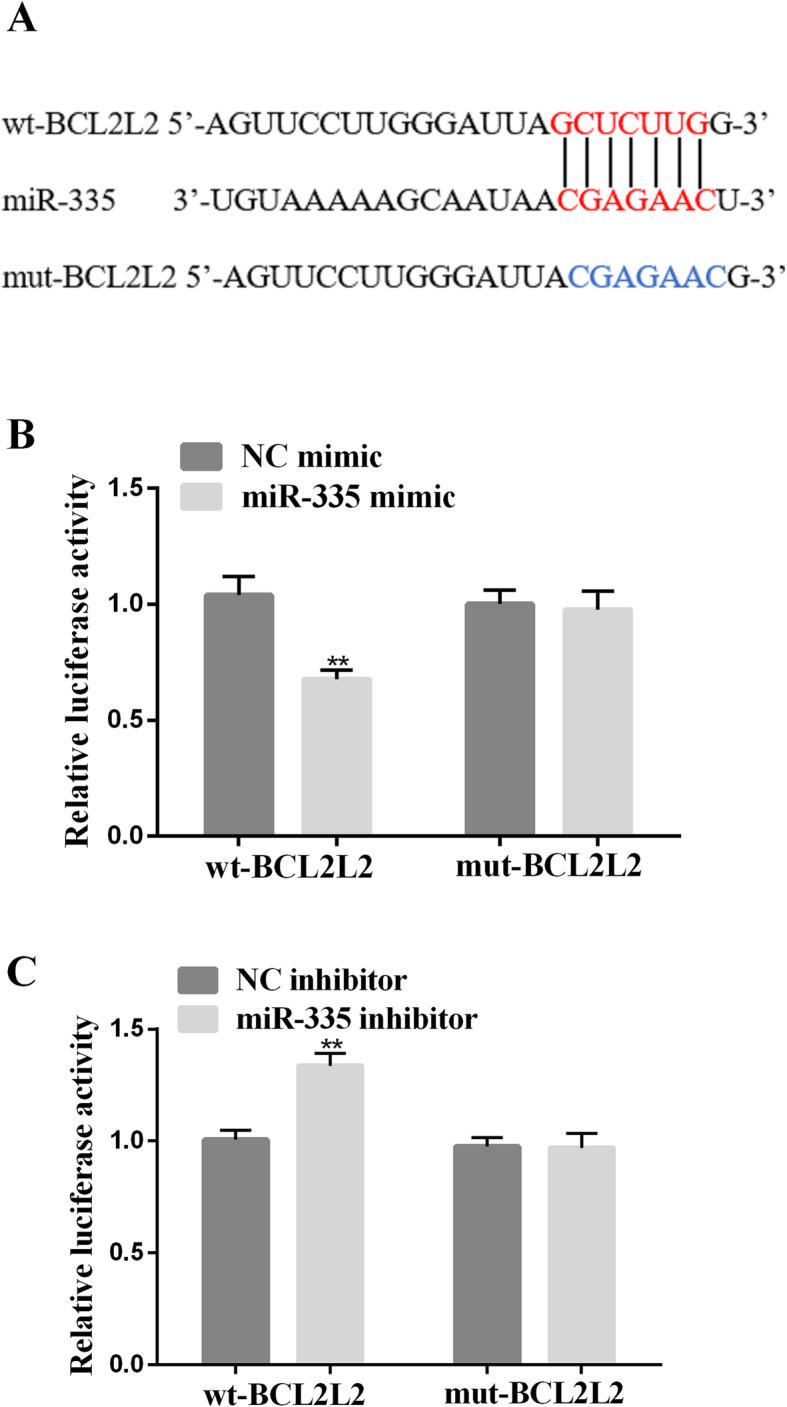
Interaction of miR-335 with 3′UTR of BCL2L2. **A** The putative miR-335 binding site in its 3′UTR contained 5-bp mutant wt-BCL2L2 and corresponding mut-BCL2L2. **B** Wt-BCL2L2/mut-BCL2L2 vector and miR-335 NC/miR-335 mimic was co-transfected into SKOV3 and OVCAR3 cells. Compared with the control group, miR-335 mimic transfection significantly reduced the luciferase activity of the wt-BCL2L2 reporter gene. In co-transfected cells with miR-335 and mut-BCL2L2 reporter genes, no significant decrease in reporter gene activity was found. **C** Wt-BCL2L2/mut-BCL2L2 vector and miR-335 NC/miR-335 inhibitor were co-transfected into SKOV3 and OVCAR3 cells. The luciferase activity of the wt-BCL2L2 reporter gene was enhanced by the miR-335 inhibitor. ** P < 0.01

We anticipated that miR-335 could combine to and target BCL2L2 3′UTR through TargetScan. The binding sites of miR-335 in wt-BCL2L2 3′UTR luciferase report vector (wt-BCL2L2) and mut-BCL2L2 3′UTR luciferase report vector (mut-BCL2L2) BCL2L2 3′UTR were created by successively mutating the predicted base pairs (Fig. 2A). Compared with the mimic NC group, the luciferase activity of the wt-BCL2L2 3′UTR luciferase reporter vector was significantly reduced in the miR-335 mimic-transfected cells. However, in miR-335 inhibitor-transfected cells, the luciferase activity was significantly increased (*p*<0.01, Fig. 2B, C).

### miR-335 inhibited the proliferation of ovarian cancer cells through BCL2L2

**Fig. 3:**
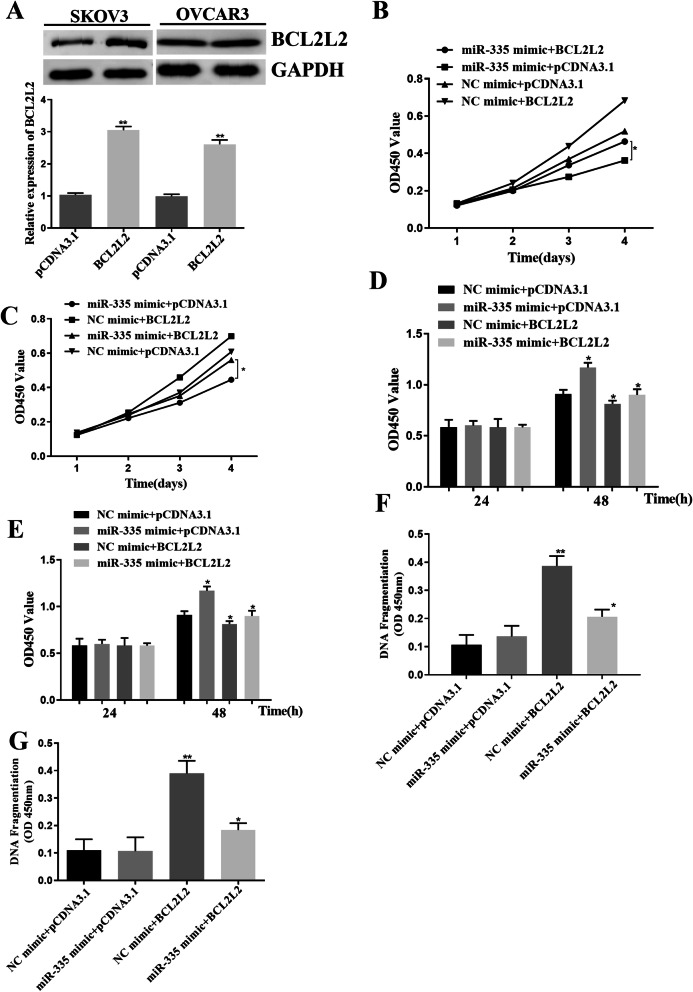
miR-335 inhibited the proliferation of ovarian cancer cell lines through BCL2L2. **A** pcDNA3.1 BCL2L2 was transfected into ovarian cancer cell lines to accomplish BCL2L2 overexpression. **B**–**E** pcDNA3.1 BCL2L2 and miR-335/NC were co-transfected into SKOV3 and OVCAR3 cell lines. The experimental results of MTT and BrdU displayed that the cell proliferation rate was obviously decreased after transfection with the mimic of miR-335, while the overexpression of BCL2L2 turned over the apparent inhibition of cell proliferation by miR-335. **F**, **G** Overexpression of miR-335 apparently advanced DNA fragmentation in SKOV3 and OVCAR3 cells, while BCL2L2 transfection weakened the influence of miR-335 on DNA fragmentation in SKOV3 and OVCAR3 cells. *P < 0.05, **P < 0.01

In order to study the function of BCL2L2 in ovarian cancer cells, we transfected pcDNA3.1 BCL2L2 into SKOV3 and OVCAR3 cell lines to overexpress BCL2L2 (Fig. 3A). We detected the level of BCL2L2 by Western blotting (*p*<0.01, Fig. 3A). Then, pcDNA3.1 BCL2L2 and miR-335 mimic/NC mimic were co-transfected into SKOV3 and OVCAR3 cell. MTT and BrdU outcome displayed that miR-335 mimic apparently decreased the amount of cell proliferation, but the overexpression of BCL2L2 saved the apparent repression of miR-335 on cell growth (*p*<0.05, Fig. 3B, C). Similarly, overexpression of miR-335 obviously promoted DNA fragmentation in SKOV3 and OVCAR3 cells. However, BCL2L2 transfection weakened the influence of miR-335 on DNA fragmentation in SKOV3 and OVCAR3 cells (*p*<0.05, Fig. 3D–G).

### XIST was related to miR-335 by direct targeting

**Fig. 4:**
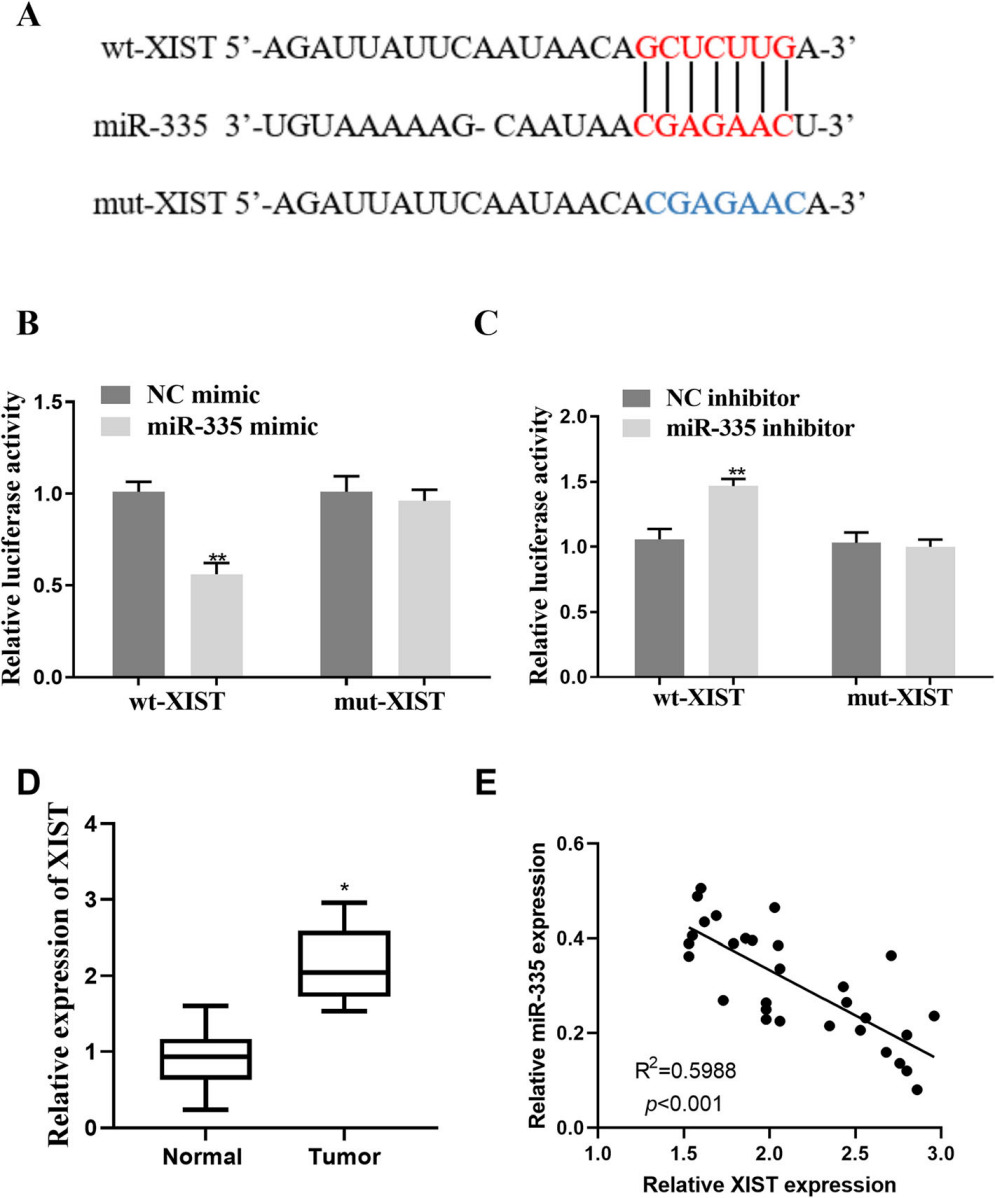
Correlation of XIST with miR-335 by direct targeting. **A** Wt-XIST 3′UTR luciferase reporter vector (wt-XIST) and mut-XIST 3′UTR luciferase reporter vector (mut-XIST) were obtained by successively mutating the calculated miR-335 combining site in XIST. **B** The luciferase activity of the wt-XIST luciferase reporter vector was significantly decreased in miR-335 cells. **C** The luciferase activity of the wt-XIST luciferase reporter vector was notably raised in the miR-335 inhibitor transfected cells. **D** XIST expression was upregulated in ovarian cancer tissues. **E** A negative correlation between XIST and miR-335 expressions in ovarian cancer tissues was identified. *P < 0.05, **P < 0.01

On the basis of former research, miR-335 has an inhibitory effect on cancer. Previous studies have reported that XIST is associated with cancer by adjusting miRNA [31]. To further evaluate the regulatory mechanism of miR-335 in ovarian tumor cell lines, we found that wt-XIST 3′UTR luciferase report vector (wt-XIST) and mut-XIST 3′UTR luciferase report vector (mut-XIST) by successively mutating the predicted miR-335 binding site in XIST 3′UTR (Fig. 4A). We co-transfected wt-XIST/mut-XIST vector and miR-335 NC/miR-335 mimic into HEK293T cells. Luciferase activity of XIST luciferase reporter vector significantly reduced compared with the NC mimic group; the expression level of miR-335 mimic-transfected cells was increased (*p*<0.01, Fig. 4B). In addition, in the miR-335 inhibitor-transfected cell group, the luciferase activity of the XIST luciferase report vector was significantly increased compared with that of the inhibitor NC group (*p*<0.01, Fig. 4C). In addition, we also found that XIST was upregulated in ovarian cancer tissues (*p*<0.05, Fig. 4C). Moreover, a negative correlation was identified between XIST and miR-335 expressions of ovarian cancer tissues (*p*<0.001, Fig. 4D).

### XIST promoted the proliferation, invasion, and migration of ovarian cancer cells

**Fig. 5:**
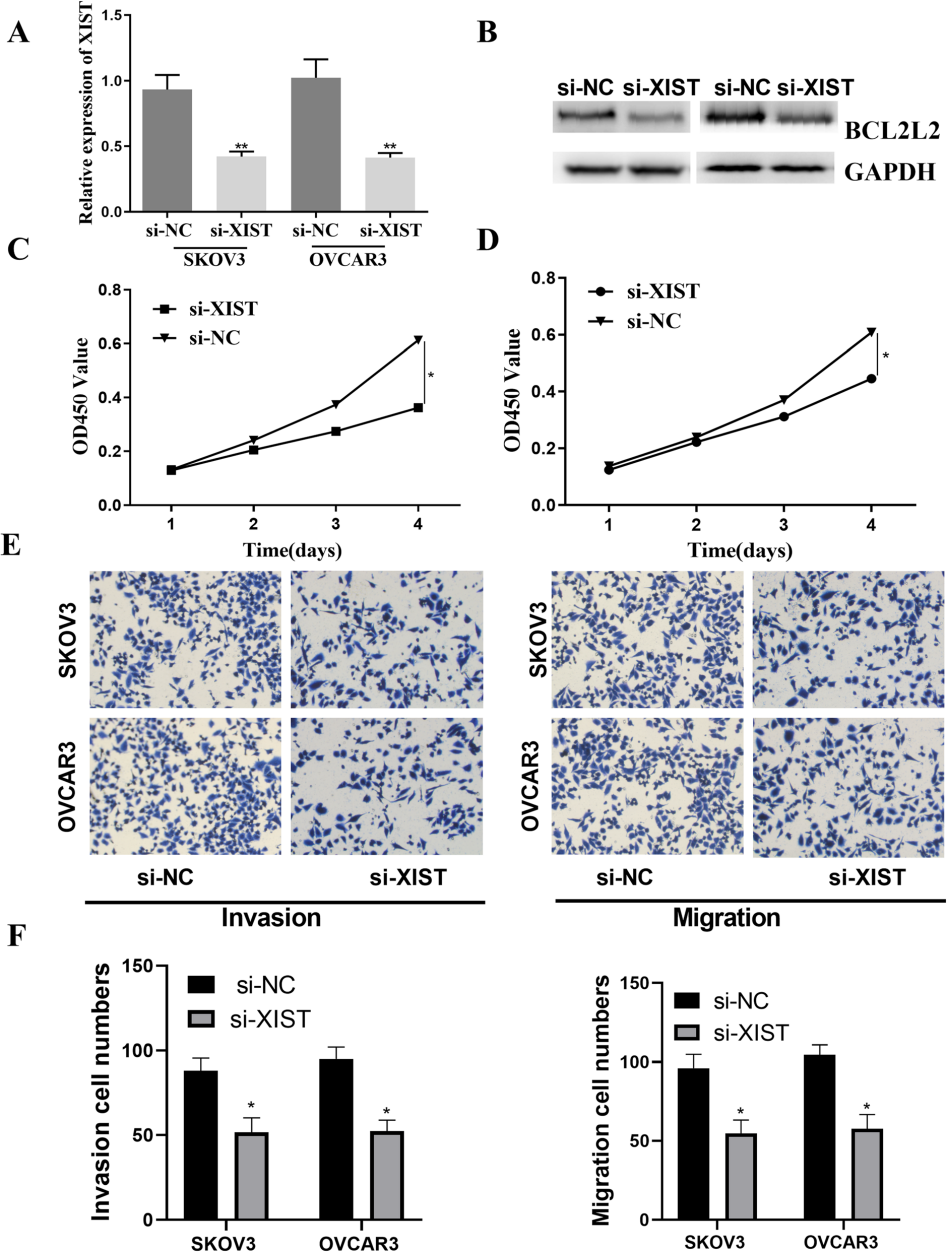
XIST silence inhibited ovarian cancer cell proliferation, invasion, and migration. **A** The XIST knockdown was achieved by si-XIST, and the inhibition efficiency was affirmed by RT-qPCR. **B** BCL2L2 expression was inhibited by si-XIST. **C**, **D** MTT assays demonstrated that the knockdown of XIST strikingly lessened the proliferation of SKOV3 and OVCAR3 cell lines over time compared with the si-NC group. **E**, **F** The invasion and migration abilities of SKOV3 and OVCAR3 cell lines were inhibited by XIST knockdown. *P < 0.05, **P < 0.01

Next, we explored the relevance between the expression of XIST and the metastasis of ovarian cancer cells. The expression of XIST was repressed by si-XIST (*p*<0.01, Fig. 5A). Moreover, the BCL2L2 expression was also inhibited by si-XIST (Fig. 5B). SKOV3 and OVCAR3 were transfected with si-NC or si-XIST, and then the proliferation was determined by MTT. MTT analysis displayed that the knockdown of XIST obviously lessened the proliferation of SKOV3 and OVCAR3 cell lines (*p*<0.05, Fig. 5C, D). Moreover, then we knock down the expression of XIST; the cell migration and invasion were apparently repressed in ovarian cancer cells (*p*<0.05, Fig. 5E, F). Finally, these records demonstrated that lncRNA-XIST could accelerate the growth, invasion, and migration of ovarian cancer cells.

### XIST promoted the expression of BCL2L2 through miR-335

**Fig. 6:**
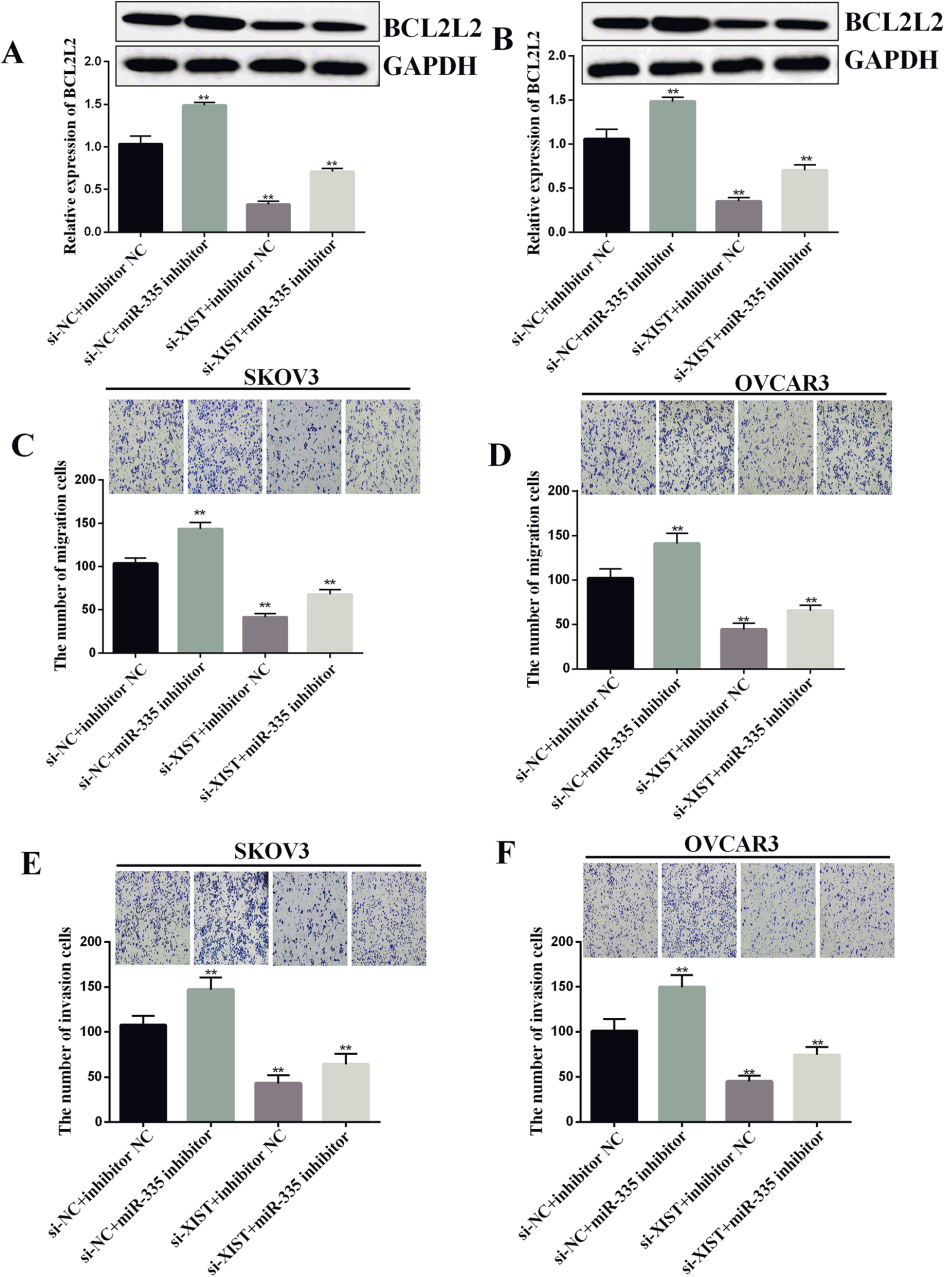
XIST promoted BCL2L2 expression through miR-335. **A**, **B** miR-335 knockdown significantly promoted the expression of BCL2L2, while si-XIST partially restored this effect in SKOV3 and OVCAR3 cell lines. **C**–**F** miR-335 inhibition significantly increased the invasion and migration of ovarian cancer cells, while simultaneous knockdown of XIST and inhibition of miR-335 partially alleviated the increase of cell invasion and migration **P < 0.01

We next studied the relationship between XIST and BCL2L2 in ovarian cancer cell lines. The outcome of qRT-PCR and Western blotting displayed that the knockdown of miR-335 obviously boosted the expression of BCL2L2, while the knockdown of XIST by si-XIST partially restored this influence in SKOV3 and OVCAR3 cell lines (*p*<0.01, Fig. 6A, B). In conclusion, XIST is most likely to promote the expression of BCL2L2 through miR-335. At the same time, we detected the cell of migration and invasion. miR-335 inhibitor promoted cell migration and invasion, but si-XIST eliminated the effect of miR-335 inhibitor (*p*<0.01, Fig. 6C–F).

## Discussion

miRNAs play important roles in almost all aspects of cancer biology, including cell growth, apoptosis, and migration [32, 33]. In this work, we focused on miR-335 as a potential tumor suppressor. Song et al. noted that overexpression of miR-335 inhibited cell migration by regulating SP1 in ovarian cancer cell lines [34]. A recent study has revealed that DANCR regulated ROCK1 via crosstalk with miR-335-5p and miR-1972. However, up to now, the role of miR-335 in the carcinogenesis of ovarian cancer and the molecular mechanism of its function are still unclear. In this study, RT-qPCR showed that the expression of miR-335 was significantly downregulated in ovarian cancer. Overexpression of miR-335 significantly inhibited the proliferation and apoptosis of ovarian tumor cells. Therefore, knocking down miR-335 can promote cell proliferation and decrease cell apoptosis. These outcomes suggest that miR-335 may be a latent tumor suppressor miRNA in ovarian cancer. The effect of particular miRNAs on cancer biology is based on their downstream targets [35, 36]. Different calculation algorithms are used to predict the gene target of miR-335, in order to clarify the potential mechanism of miR-335 induced inhibition of ovarian cancer growth and metastasis. BCL2L2 oncogene is frequently overexpressed in many malignant tumors, and as an important regulator of cell proliferation, survival, and metastasis, it has been identified as a key downstream target of miR-335 [37–39].

In this study, overexpression of miR-335 downregulated the protein level of BCL2L2. At the same time, knockdown miR-335 upregulated the expression of BCL2L2. The binding and inhibition of miR-335 on BCL2L2 3′UTR in SKOV3 and OVCAR3 cells were confirmed by luciferase reporter gene assay. The results of Western blot also confirmed that miR-335 could regulate the expression of BCL2L2. In addition, the expression level of miR-335 and BCL2L2 in clinical ovarian cancer samples was significantly negatively correlated. In order to further confirm the role of BCL2L2 in SKOV3 and OVCAR3 cell, we manifested that the inhibition of BCL2L2 can inhibit the proliferation of SKOV3 and OVCAR3 cell line and promote its apoptosis. These data on the involvement of the miR-335/BCL2L2 axis in ovarian cancer demonstrate that miR-335 might have the potential as a therapeutic target.

In order to identify the interaction between miRNA and lncRNA, its potential mechanism provides a potential target for tumor therapy. Previous studies focused on the target of miRNA and the mechanism of miRNA regulatory target, thus affecting the occurrence and development of tumors. For instance, miRNAs containing miR-21, miR-34a, and miR-182 [40–42] are reported to participate in tumor growth and development. However, little attention has been paid to the control of miRNA, especially the control of miRNA by non-coding RNA. Emerging evidence suggests that the interaction between miRNA and lncRNA plays a major role in tumor progression. In this study, we first revealed the interaction between XIST and miR-335. The knockdown of XIST upregulated miR-335, while the overexpression of forced miR-335 inhibited the expression of XIST.

To detect the influence of XIST in the growth regulation of ovarian cancer cells, siRNA was transfected into ovarian cancer cells to knock down XIST. As speculated, BCL2L2 was downregulated by XIST. According to previous studies, the expression of BCL2L2 can regulate the apoptosis of cardiomyocytes [43]. In order to further verify the connection of XIST, miR-335, and BCL2L2 in ovarian cancer, the levels of XIST, miR-335, and BCL2L2 were measured. The results showed that the levels of XIST and BCL2L2 were upregulated, while miR-335 was downregulated.

## Conclusion

In a word, XIST can inhibit the activity of ovarian cancer cells by regulation of BCL2L2 and miR-335. The XIST/miR-335/BCL2L2 axis determined in our study might provide potential treatment strategies for ovarian cancer.

## Data Availability

The datasets used and/or analyzed during the current study are available from the corresponding author on reasonable request.
